# Increased levels of nectin-4 as a serological marker for pre-eclampsia

**DOI:** 10.20407/fmj.2022-027

**Published:** 2022-12-27

**Authors:** Hikari Yoshizawa, Haruki Nishizawa, Mayuko Ito, Akiko Ohwaki, Yoshiko Sakabe, Takao Sekiya, Takuma Fujii, Hiroki Kurahashi

**Affiliations:** 1 Department of Obstetrics and Gynecology, Fujita Health University, School of Medicine, Toyoake, Aichi, Japan; 2 Division of Molecular Genetics, Institute for Comprehensive Medical Science, Fujita Health University, Toyoake, Aichi, Japan

**Keywords:** *NECTIN4*, Placenta, Pre-eclampsia

## Abstract

**Objectives:**

Nectin-4 is a cell adhesion molecule with vital functions at adherens and tight junctions. Cumulative evidence now indicates that the *NECTIN4* gene is overexpressed in a variety of cancers, and that the nectin-4 protein is both a disease marker and therapeutic target in a subset of these cancers. We previously demonstrated that *NECTIN4* is overexpressed in placenta during pre-eclamptic pregnancy, which is one of the most serious obstetric disorders.

**Methods:**

Nectin-4 protein levels were measured in maternal sera from pregnant women with pre-eclampsia and its related disorder, unexplained fetal growth retardation.

**Results:**

Maternal serum concentrations of nectin-4 were significantly elevated in pre-eclamptic women compared with those with an uncomplicated normotensive pregnancy. However, no increase was observed in pregnancies with unexplained fetal growth retardation. Serum nectin-4 levels were higher in cases with early-onset pre-eclampsia that generally showed more severe clinical symptoms, but levels were not correlated to other clinical indicators of disease severity.

**Conclusions:**

Nectin-4 is a potential new diagnostic and predictive biomarker for severe pre-eclampsia.

## Introduction

The nectin-4 protein was originally described as a homolog of poliovirus receptor-like receptors.^1^ Nectins are known to be Ca^2+^-independent, immunoglobulin-like cell adhesion molecules that act mainly at both adherens and tight junctions.^2^ Although nectin-1, -2 and -3 have been well characterized as adhesion molecules, the functions of nectin-4 have remained unknown to date. Certain cancer cells or cell lines, such as breast, lung and ovarian cancers, have been found to overexpress nectin-4.^3–5^ Nectin-4 is also known to be cleaved by a disintegrin and metalloproteinase (ADAM) proteases and shed into the bloodstream.^6^ Indeed, soluble forms of nectin-4 have been identified at high levels in the serum of cancer patients, and it has been suggested as a new biomarker of these cancer types.^3,4,6,7^ More recently, nectin-4 has exhibited characteristics as a potential target for cancer therapy. Enfortumab vedotin, an anti-nectin-4 antibody-drug conjugate, has shown microtubule-disrupting properties and been reported as an encouraging new treatment approach for urothelial tumors.^8,9^

Pre-eclampsia is one of the most common obstetrical complications, accounting for almost 15% of pregnancy-associated disorders. However, although it is principally defined by the onset of hypertension with proteinuria,^10^ it is not a simple disease but a syndrome of multiple organ failure involving the liver, kidneys and lungs, in addition to coagulation and neural system difficulties. Since severe pre-eclampsia has a considerably poor prognosis for both mother and fetus, it is potentially one of the most serious pregnancy-associated disorders. Although a recent study demonstrated that the reduction of circulating placental growth factor is a sufficiently sensitive marker to enable the pre-symptomatic diagnosis of pre-eclampsia, a scarcity of predictive biomarkers for severe pre-eclampsia has still hindered the timely prevention and treatment of this disorder.^11,12^

The *NECTIN4* gene is known to be expressed most abundantly in the placenta^13^ and we previously showed it was significantly upregulated in pre-eclampsia.^14,15^ Further, we recently demonstrated that the nectin-4 protein is overexpressed at the apical cell membrane of syncytiotrophoblasts in placentas from patients with pre-eclampsia, and that nectin-4 overexpressing trophoblast cells induce strong cytotoxic effects in NK cells, suggesting a possible etiologic role of nectin-4 in this disease.^16^ Localization of nectin-4 also suggests the possibility of its detection in the maternal circulation. In the current study, we determined the concentration of serum nectin-4 in women with severe pre-eclampsia as well as its related disorder, unexplained fetal growth retardation (FGR), in comparison with uncomplicated normotensive pregnancies. We discuss the utility of nectin-4 as a potential biomarker of pre-eclampsia, as well as its etiological role in this disorder.

## Subjects and Methods

### Subjects

All blood samples analyzed in this study were collected at the Department of Obstetrics and Gynecology, Fujita Health University Hospital, Japan. Severe pre-eclampsia was defined as a blood pressure greater than 160/110 mmHg, and proteinuria of more than 2 g in a 24 h collection (*n*=41), although this criteria was revised and the latest version is currently used.^17,18^ FGR was diagnosed when the birth weight was below the 10th percentile of that anticipated for the given gestational age in the Japanese population (*n*=20).^19^ Unexplained FGR was defined by the exclusion of known maternal and fetal factors, including maternal systemic diseases, multiple gestation, fetal congenital infection, structural abnormalities and chromosomal abnormalities. All of the unexplained FGR cases in our current study series showed clinical signs of disturbed placental function, such as asymmetric growth, oligohydramnios, and/or an increased pulsatility index of the umbilical artery. Normotensive subjects were matched for maternal age and body mass index during pre-pregnancy (*n*=48). The clinical details of these subjects are presented in Table 1. Additionally, samples were also obtained from normotensive uncomplicated pregnancies at the 1st trimester of gestation (*n*=18). Serum samples were stored at –80°C until use. Informed consent was obtained from each participant and this study was approved by the Ethical Review Board for Clinical Studies at Fujita Health University.

### Enzyme-linked immunosorbent assay (ELISA)

The serum nectin-4 concentrations were measured using a commercially available Quantikine^®^ ELISA kit, Human Nectin-4 Immunoassay (R&D Systems, Minneapolis, MN), in accordance with the manufacturer’s instructions. All samples were run in duplicate. The intra- and inter-assay coefficients of variation were less than 3.83% and 5.90%, respectively. The detection limit for recombinant nectin-4 was approximately 39.1 pg/ml. The calibration was linear up to 2500 pg/ml.

### Statistical analysis

Intergroup comparisons were made using the Mann–Whitney U test or one way analysis of variance method, and *P* values less than 0.05 were considered statistically significant. Correlations were evaluated using a Spearman’s test. For significant difference tests, *P* values were calculated with the z conversion of Fisher’s r, and those less than 0.05 were considered statistically significant. Receiver operating characteristic curves were plotted to investigate the predictive value of serum nectin-4 for severe pre-eclampsia. Youden’s index (sensitivity+specificity–1) was also used to determine the optimal cut-off value of serum nectin-4 levels from the receiver operating characteristic plot.^20^

## Results

**Figure 1:**
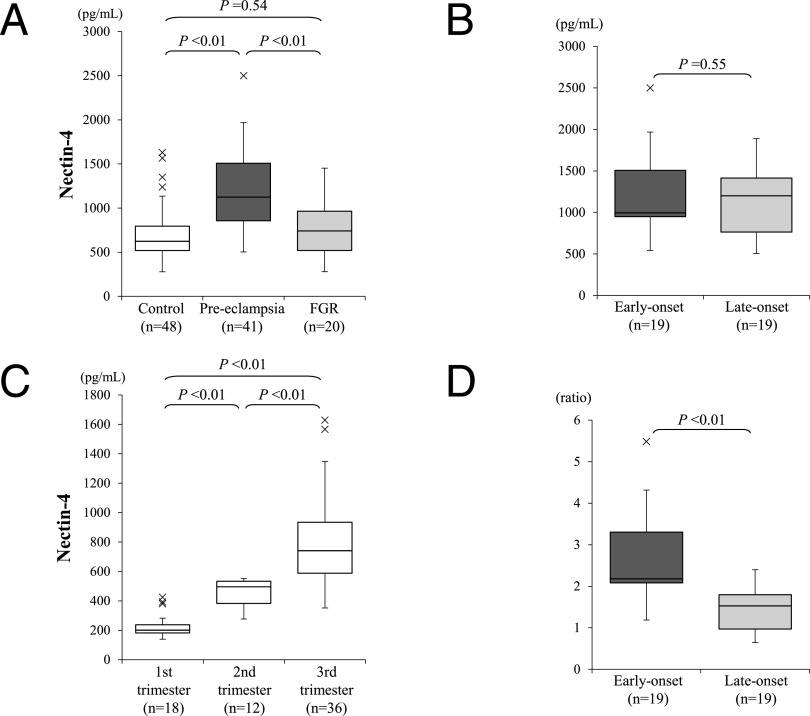
Serum concentrations of nectin-4 measured by ELISA. **A.** High serum nectin-4 concentrations were present in serum samples from patients with pre-eclampsia. These levels were compared between the sera from cases with an uncomplicated normotensive pregnancy (left), pre-eclampsia (center) and unexplained fetal growth retardation without hypertension (right). The boxes indicate the 25th and 75th percentiles, and bands near the middle indicate median values. The bars indicate 1.5 interquartile ranges with the outliers specifically marked. **B.** Correlation between serum nectin-4 levels and disease onset. **C.** Correlation between nectin-4 levels and gestational week in normotensive uncomplicated pregnancies throughout gestation. **D.** Correlation between the corrected serum nectin-4 levels, divided by the mean value of serum nectin-4 from uncomplicated normotensive controls matched for gestational week.

Serum nectin-4 levels were detected at significantly higher levels in patients with pre-eclampsia than in control women with uncomplicated normotensive pregnancies (Figure 1A). This assay showed the potential utility of nectin-4 in the diagnosis of pre-eclampsia. In receiver operating characteristic curve analysis, the area under the curve was 0.833. The cut-off value for severe pre-eclampsia of 838 pg/ml nectin-4 allowed for a sensitivity of 78.0% and specificity of 77.1% at the optimal cut-off point, while the cut-off value of 948 pg/ml nectin-4 allowed for sensitivity of 70.7% and specificity of 85.4% in accordance with the Youden index criteria. We also determined the levels of nectin-4 protein in maternal sera from cases with the pre-eclampsia-related disorder unexplained FGR, but found no elevation in these cases (Figure 1A, right).

To examine the potential utility of nectin-4 as a reliable disease marker of severe pre-eclampsia, we determined whether its serum levels correlated with the severity of this disorder. Nineteen patients with early-onset (earlier than 34 weeks gestation) and 19 patients with late-onset (34 weeks gestation or later) disease were compared, but no significant differences were observed (Figure 1B). However, in subjects with uncomplicated normotensive pregnancy, serum nectin-4 levels were low in early gestation and then significantly increased with gestational age (1st trimester, n=18; 2nd trimester, n=12; 3rd trimester, n=36; Figure 1C). Thus, uncomplicated normotensive 2nd trimester pregnancy (Figure 1C, center) was used as a control for early-onset pre-eclampsia (28.3 versus 31.0 mean gestational weeks, respectively), and normotensive 3rd trimester pregnancy (Figure 1C, right) was used as a control for late-onset pre-eclampsia (37.5 versus 36.0 mean gestational weeks, respectively). Comparison of serum nectin-4 levels that had been adjusted using data from gestational age-matched uncomplicated normotensive pregnancy controls showed significantly higher nectin-4 levels in the early-onset pre-eclamptic group (Figure 1D).

**Figure 2:**
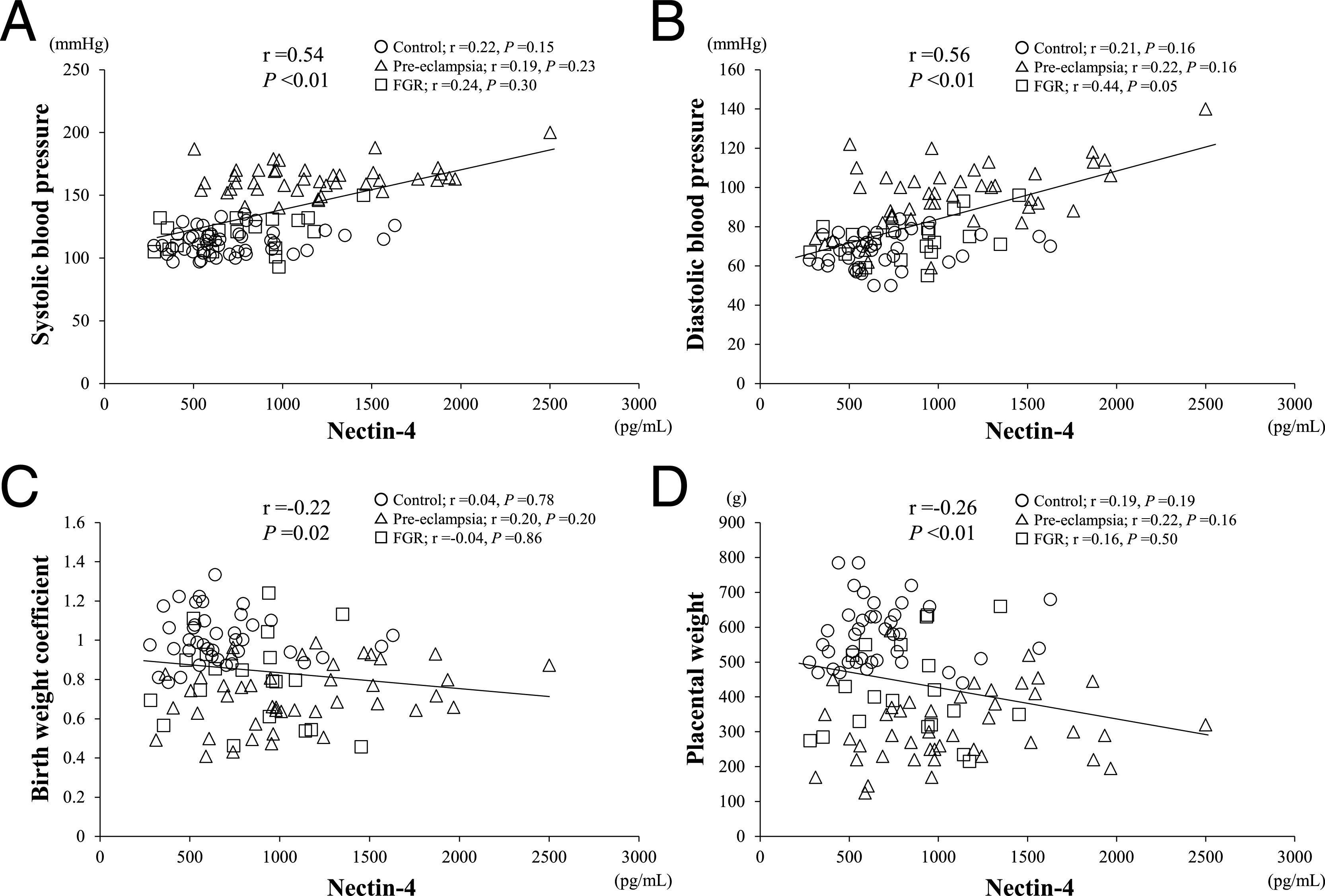
Analysis of the correlations between the serum concentrations of nectin-4 and various clinical parameters associated with pre-eclampsia. **A.** Systolic blood pressure. **B.** Diastolic blood pressure. **C.** Normalized birth weight. **D.** Placental weight. Open circles indicate control uncomplicated pregnancies, open triangles indicate pre-eclampsia and open boxes indicate fetal growth retardation. Regression lines are shown with correlation coefficients and *P* values.

To assess the possible association between the serum nectin-4 concentrations and the severity of pre-eclampsia, this study determined whether maternal serum nectin-4 levels were correlated with other clinical parameters. A positive correlation was found with both systolic and diastolic blood pressure (Figure 2A, B). However, no correlation was observed when these analyses were performed separately for the three groups; i.e., the pre-eclampsia, unexplained FGR and normotensive control populations. This finding indicated that the correlation between nectin-4 levels and blood pressure simply reflected the presence of pre-eclampsia and not its severity.

A similar observation was made for the association between birth and placental weights. Normalized birthweights were calculated by dividing the measured weight by the expected standard weight at a particular gestational week in the general Japanese population,^19^ prior to the correlation assessments. Maternal serum nectin-4 levels showed a significant inverse correlation with normalized birth weight in our study cohort (Figure 2C). However, no correlation was observed when analyses were performed separately for the pre-eclampsia, unexplained FGR and control groups. This finding suggested that the correlation between nectin-4 levels and birth weight outcomes simply reflected the presence of the disease. Similar to the data obtained for birthweight outcomes, maternal serum nectin-4 levels were inversely correlated with placental weight (Figure 2D), but again, this correlation was not observed when analyses were performed separately for the three groups.

## Discussion

The present study revealed that patients with severe pre-eclampsia have significantly elevated circulating nectin-4 levels. Several proteins have been shown to be elevated in the maternal sera of patients with pre-eclampsia, including activin A/inhibin A, leptin, soluble Fms-related tyrosine kinase and endoglin.^21,22^ We previously screened for upregulated genes in placentas from cases with pre-eclampsia, and proposed the usefulness of measuring serum pregnancy-associated plasma protein A2 and high temperature requirement protease A4 levels as candidate biomarkers for this condition, based on good sensitivity and specificity levels.^23,24^ Using a similar strategy, the current findings have identified nectin-4 as an additional potential biomarker for pre-eclampsia. To date, nectin-4 has been studied as a potential biomarker mainly in the field of cancer research, with a demonstrated overexpression in a number of tumor types, including breast, lung, urothelial, colorectal, pancreatic and ovarian cancer.^8,25^ Serum nectin-4 concentrations were shown to be elevated in patients with these cancers. The current study is the first report of high serum nectin-4 levels in a non-cancer disorder.

Serum nectin-4 levels showed good sensitivity and specificity in detecting pre-eclampsia in our current evaluations. To further evaluate its utility as a diagnostic and prognostic marker for this disorder, we analyzed its association with key clinical pre-eclamptic parameters that reflect the severity of this disease, such as systolic and diastolic blood pressure, and birth and placental weights. Although correlations were observed between serum nectin-4 concentrations and these parameters when all pre-eclamptic subjects were analyzed together, no such association was evident when analyses were performed separately for the pre-eclampsia and normotensive control groups. This lack of association suggests that the observed correlation reflects the presence of the disease but not its severity.

Among the examined parameters in the current study, only the onset of pre-eclamptic symptoms showed an association with serum nectin-4 levels when corrected for the gestational week. Although patients with early-onset pre-eclampsia often develop more acute symptoms, the fact that other clinical parameters tested in the current analyses did not correlate with serum nectin-4 levels suggested that a higher concentration of this protein in early-onset cases does not reflect disease severity. Although the diagnosis of severe pre-eclampsia is readily achievable by current clinical assessments, such as blood pressure, clinicians are still awaiting the development of early biomarkers of this disorder that can be used to predict the subsequent development of severe pre-eclampsia in apparently uncomplicated pregnancies. In this regard, circulating placental anti-angiogenic or angiogenic factors, such as soluble fms-like tyrosine kinase-1 or placental growth factor, are known to potentially predict the onset of pre-eclampsia. A recent study reported that a reduction in circulating placental growth factor levels was sufficiently sensitive to enable the pre-symptomatic diagnosis of pre-eclampsia.^12^ Since nectin-4 levels were significantly higher in early-onset disease cases in the current investigation, future prospective randomized studies should help to clarify its potential usefulness for the early detection of pre-eclampsia.

The observed correlations between serum nectin-4 levels and the timing of disease onset suggests possible etiological heterogeneity in pre-eclampsia. It has been well documented that pre-eclampsia is a polygenetic disease triggered by multiple genetic (maternal and fetal) and environmental factors. A two-stage disease hypothesis has been proposed, in which an initiating reduction of placental perfusion by abnormal vascular remodeling leads to maternal symptoms in individuals who have a genetic predisposition to this disease.^26^ With regard to fetal or placental factors in this disorder, there is the possible involvement of abnormal placentation or altered expression of soluble placental proteins in the initial etiology of pre-eclampsia. It is likely that early-onset pre-eclampsia has a different etiology from the late-onset disease. Early-onset pre-eclampsia is recognized as a placental disease, featuring vascular lesions with a reduced blood supply.^27^ The current data showing higher levels of circulating nectin-4 in cases with early-onset pre-eclampsia, combined with its abundant expression in villous trophoblasts in the pre-eclamptic placenta, suggest that upregulation of nectin-4 expression may be involved in the etiology of pre-eclampsia, particularly in early-onset disease.^13,16^

It is noteworthy that a growing body of evidence indicates a pivotal function of nectin-4 in natural immunity.^28^ Nectin-4 was originally described as a homolog of poliovirus receptor-like receptors, and was later found to be an epithelial receptor for the measles virus.^1,29^ It has been proposed that viral-infected cells undergo the induced expression of cluster of differentiation 155 and nectin-4 proteins, which are ligands of DNAX accessory molecule-1 and TIGIT (T cell immunoreceptor with Ig and ITIM domains) proteins, paired activating and inhibitory receptors, respectively, in NK cells that modulate host-pathogen interactions.^28^ A recent study found a significant role for nectin-4 during NK cell activity against the SARS-CoV-2 virus.^30^ We have previously demonstrated that nectin-4 overexpressing villous trophoblast cells induce strong cytotoxic effects from NK cells.^16^ This observation, combined with the high concentration of serum nectin-4 in patients with early-onset pre-eclampsia, suggests that alterations to immune reactivity at the feto-maternal interface of the placenta in early gestation may contribute to the etiology of pre-eclampsia. This novel hypothesis deserves further investigation.

## Figures and Tables

**Table1:** Clinical parameters of the study groups

	Control n=48	PE n=41	FGR n=20	*P* Value		
				Control vs PE	Control vs FGR	PE vs FGR
Maternal age (y)	31.2±4.6^†^	31.3±4.8	31.3±4.2	n.s.*	n.s.	n.s.
Gestational age (weeks)	35.2±4.0	33.8±3.1	35.2±2.4	n.s.	n.s.	n.s.
Systolic BP (mmHg)	113.0±10.0	163.0±12.0	118.6±14.0	<0.01	n.s.	<0.01
Diastolic BP (mmHg)	67.7±8.2	101.0±12.0	75.2±10.5	<0.01	n.s.	<0.01
Proteinuria^‡^	0 (0%)	41 (100%)	0 (0%)	<0.01	n.s.	<0.01
Body mass index^§^	20.9±2.9	22.1±4.0	20.8±2.9	n.s.	n.s.	n.s.
Birth weight (g)	2997.6±345.6	1616.1±611.7	1585.8±566.7	<0.01	<0.01	n.s.
Birth weight coefficient^#^	1.013±0.127	0.748±0.135	0.616±0.146	<0.01	<0.01	n.s.
Placental weight (g)	576.1±88.1	325.9±99.0	302.8±89.9	<0.01	<0.01	n.s.

^†^ Data are mean values±standard deviation. * n.s.: not significant. ^‡^ ≥2 g in a 24 h collection. ^§^ pre-pregnancy. ^#^ The birthweight coefficient was calculated by dividing the measured birthweight by the expected standard birthweight at that gestational week. FGR: fetal growth restriction, PE: pre-eclampsia.
